# Rituximab Was Effective in Relieving Symptoms of Isaacs Syndrome: A Case Report

**DOI:** 10.7759/cureus.30100

**Published:** 2022-10-09

**Authors:** Kazuhiro Horiuchi, Akihiko Kudo, Takashi Inoue, Shintaro Fujii, Yuki Oshima

**Affiliations:** 1 Neurology, Hakodate Municipal Hospital, Hakodate, JPN; 2 Neurology, Faculty of Medicine and Graduate School of Medicine, Hokkaido University, Sapporo, JPN

**Keywords:** plasma exchange, intravenous immunoglobulin, myokymia, rituximab, neuromyotonia, isaacs syndrome

## Abstract

We presented a 23-year-old patient who had experienced neuromyotonia in his left leg. Although he tested negative for anti-LGI1 and anti-CASPR2 antibodies, we diagnosed him with Isaacs syndrome due to myokymic discharges on electromyography and symptoms being relieved by intravenous methylprednisolone (IVMP) and intravenous immunoglobulin (IVIg). IVMP, IVIg, plasma exchange, or cyclosporine treatment did not provide a long-term response; however, rituximab showed long-term improvement. Rituximab should be considered early in the treatment of patients with antibody-negative Isaacs syndrome who are responsive to immunotherapy, including IVMP, IVIg, and plasma exchange, and have long-term symptoms that are hard to control.

## Introduction

Isaacs syndrome is a disorder characterized by painful muscle stiffness and spasm, myokymia, and peripheral nerve hyperexcitability (PNH) [1]. Presently, autoantibodies are identified in 45%-50% of patients with Isaacs syndrome [1,2]. These are antibodies against VGKC complex proteins such as contactin-associated protein 2 (CASPR2), leucine-rich glioma inactivated 1 (LGI1), and contactin 2 [3,4]. However, the absence of specific antibody targets in the remaining patients suggests that more autoantibody targets are yet to be discovered [5].

Membrane stabilizers, such as carbamazepine, are effective in relieving symptoms of Isaacs syndrome. Immunotherapy, including plasma exchange, intravenous immunoglobulin therapy (IVIg), and high-dose intravenous methylprednisolone (IVMP) therapy, has also been effective in the treatment of Isaacs syndrome. However, some patients are refractory to treatment [2].

Herein, we report a case of Isaacs syndrome associated with extremely painful muscle spasms in the left leg, in whom rituximab was effective in relieving symptoms.

## Case presentation

The patient was a 23-year-old man with no remarkable past medical history. His family history was also unremarkable. He had experienced spasm-like involuntary movements and pain in his left thigh muscle three days earlier. He was treated with eperisone at another hospital; however, because this treatment was ineffective, he came to our department because of persistent involuntary movements and severe pain in his left thigh muscle.

On physical examination, he was in anguish. He had a pulse rate of 87 beats/min; blood pressure of 123/72 mmHg; oxygen saturation of 98%; and temperature of 37.6°C. He was 170 cm tall and weighed 74 kg. His eyes were not congested. His breathing sounds were normal on chest auscultation, his abdomen was not tender, and there was no body edema. During a neurological examination, he was alert, and his mental state and cranial nerve findings were normal. His upper extremity muscle strength and deep tendon reflexes of his extremities were normal, and his Babinski and Chaddock reflexes were negative. No sensory deficits were noted. The muscle strength of the proximal muscles of the left lower extremity (iliopsoas, quadriceps femoris, and hamstring) was 4 by manual muscle testing. He complained of pain in all his left leg, especially in the femoral muscles. His left femoral muscles showed stiffness, hyperhidrosis, muscle contractions, and myokymia (fine quivering, rippling, and undulating contractions), and his left toe movement showed grip myotonia (muscles were not able to relax after contracting). He had difficulties walking but no percussion myotonia. His symptoms were localized to the left leg and persisted during sleep.

Peripheral blood tests showed normal creatine kinase (CK) levels (47 U/L). Serum levels of thyroid hormone, lactic acid, and pyruvic acid were within normal ranges. Anti-acetylcholine receptor (AchR) antibody, anti-glutamic acid decarboxylase antibody, anti-CASPR2 antibody, and anti-LGI1antibody tests were negative. The anti-Contactin2 antibody level was not measured. During the cerebrospinal fluid (CSF) test, the lumbar CSF opening pressure was 170 mmH2O, the number of cells was 4/μL (lymphocytes: 4, neutrophils: 0), the protein level was 21 mg/dL, glucose level was 63 mg/dL, IgG level was 19.0 mg/dL, IgG index was 0.49, and myelin basic protein level was <40 mg/dL. The oligoclonal band was positive.

Chest radiographs and computed tomography (CT) images of his body were normal, and no thymoma was observed. Brain and spinal cord magnetic resonance imaging (MRI) findings were normal. MRI on short inversion time inversion recovery imaging of the proximal lower limbs showed no intensity changes.

**Figure 1:**
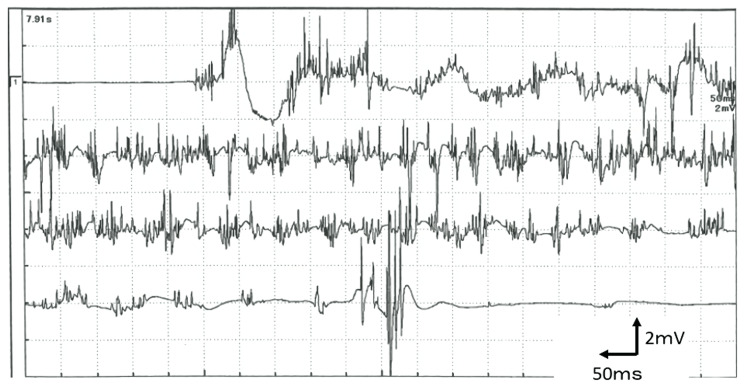
Electromyography at the rectus femoris muscle Electromyography showing the abnormal repetitive spontaneous activity of the muscle fibers (doublet, triplet, or multiple single-unit discharges with a high, irregular intraburst frequency) at the rectus femoris muscle

Electroencephalography findings were normal. Needle electromyography (EMG) revealed continuous motor unit activity with myokymic discharges at the rectus femoris muscle (Figure 1). Nerve conduction studies were normal. No stimulus-induced repetitive discharge was detected. Given his set of symptoms, our suspected diagnosis was neuromyotonia due to focal Isaacs syndrome of the left leg. We decided to make a diagnosis after treating him.

**Figure 2:**
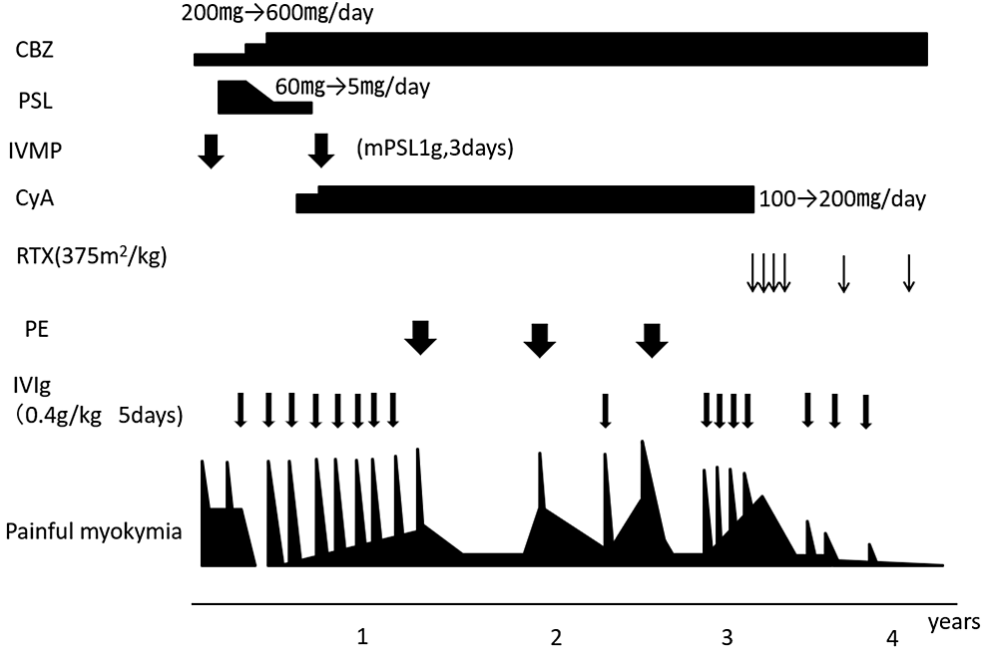
The clinical course of the patient CBZ: carbamazepine, PSL: prednisolone, IVMP: intravenous prednisolone, mPSL: methylprednisolone, CyA: cyclosporine A, RTX: rituximab, PE: plasma exchange, IVIg: intravenous immunoglobulin

His clinical course is shown in Figure 2. He was initially given 200 mg/day of carbamazepine, which slightly improved the pain in the left lower leg; however, the myokymia persisted. Subsequently, intravenous methylprednisolone (IVMP) was administered at 1000 mg/day for three days, followed by a gradual decrease in the dose of prednisolone from 60 mg/day. Immediately after the administration of 1000 mg of methylprednisolone, the myokymia and pain in the left leg resolved; however, the symptoms worsened within a few days of switching to oral prednisolone. Therefore, prednisolone was tapered off. Because the pain persisted, tramadol and baclofen were tried; however, they were ineffective. Intravenous immunoglobulin (IVIg: 0.4 g/kg, 5 days) was administered due to persistent pain and myokymia. The symptoms in the left lower leg improved markedly after the start of the treatment, after which the patient was discharged from our hospital. Although the patient tested negative for anti-LGI1 and anti-CASPR2 antibodies, he was found to have neuromyotonia (myotonia of peripheral nerve origin; clinically, grip myotonia but no percussion myotonia), persistent muscle cramps and muscle stiffness in the left leg that even during sleeping, myokymic discharges on electromyography that showed hyperexcitation of the peripheral nerves, and symptoms that were relieved by IVMP and IVIg administration. We diagnosed him with Isaacs syndrome after the treatment.

Two weeks after being discharged from the hospital, the painful myokymia of his left leg worsened; so, we administered pregabalin and increased the dose of carbamazepine; however, their curative effect was limited. We treated him with IVIg again, and his symptoms improved. Painful myokymia improved after IVIg but quickly worsened within a few weeks. IVMP was tried again but had little effect. Therefore, IVIg was given once every two months. The patient improved after receiving IVIg but gradually became less effective. Cyclosporine was also tried as an immunosuppressive agent but failed to prolong the duration of symptom relief. His symptoms had worsened but were limited to the left leg. Eighteen months after the initial hospitalization, we initiated plasma exchange (albumin replacement, five times in total). The plasma exchange was effective, and the patient's symptoms remained stable for about six months but flared up again thereafter; so, we repeated the plasma exchange. Symptoms were relieved for six months after the first plasma exchange but then worsened approximately three months after the second plasma exchange. However, during the third plasma exchange, a thrombus developed in the right internal jugular vein, resulting in thrombophlebitis and the occlusion of the vein. Since he did not accept repeat plasma exchange with the placement of a catheter at another site, we decided to avoid further plasma exchange. IVIg was administered monthly every time his symptoms worsened.

Rituximab was started because monthly IVIg was not sufficiently effective. Rituximab was administered at 375 mg/m^2^ weekly for a total of four doses. After rituximab administration, the myokymia in the left lower extremity began to improve, and most of the severe pain resolved. Before rituximab, IVIg was administered monthly every time his symptoms worsened; however, it became necessary to treat it every three months, and then every six months thereafter. Four years after the initial hospitalization, the administration of intravenous globulin was no longer necessary.

Four years after the initial hospitalization, rituximab, at a dose of 375 mg/m^2^, was continued every six months. The myokymia in the left leg has almost disappeared. Other than mild leukopenia (WBC 3000/mm^3^), no other side effects of rituximab (including infections) have been observed. Opioids were started for the residual pain in the left lower leg, which could be controlled with a fentanyl patch at a dose of 1 mg/day. The pain in his left leg reduces when the patient is at rest. He requires a wheelchair for mobility because he feels pain when he stands on his left leg.

CT scans taken at intervals over the course of four years revealed no malignant tumors (including thymoma).

## Discussion

In approximately 40% of cases, patients with Isaacs syndrome test positive for anti-VGKC complex antibodies, including the CASPR2 and LGI1 antibodies [5]. In this case, the patient tested negative for the CASPR2 and LGI1 antibodies. However, we found his signs and symptoms to be consistent with those of Isaacs syndrome because he showed neuromyotonia with severe pain due to PNH, accompanied by needle electromyography findings and the fact that improvement was achieved with IVMP, IVIg, and plasma exchange. His symptoms were limited to the left leg; therefore, we diagnosed him with focal-type Isaacs syndrome. Neuromyotonia limited to the gastrocnemius muscle only or to the lower part of the leg has been reported previously [6,7].

The treatment of Isaacs syndrome generally involves symptomatic treatment with sodium channel inhibitors, such as carbamazepine and phenytoin, to suppress PNH. Diazepam, which acts on the central nervous system, is considered ineffective. Furthermore, because the symptoms are caused by immune-mediated mechanisms, IVIg, plasma exchange, steroids, tacrolimus, cyclosporine, and rituximab have been found to be effective [8]. In certain cases, some drugs are effective in relieving symptoms, whereas others are not. Antiepileptic drugs were given to 15 of 17 patients, steroids to six of 12, and plasma exchange was performed in eight. Immunosuppressive treatments, such as IVIg, tacrolimus, cyclosporine, and rituximab, have been reported to be effective in 14 of 17 patients [2]. Three positive anti-CASPR2 antibody-positive cases were reported with favorable results with corticosteroids, double-filtration plasmapheresis, and rituximab [9]. Although patients with symptoms of Isaacs syndrome as a paraneoplastic syndrome were more severely ill than those without tumors, they also responded better to treatment [2].

There is a strong association with cancer, with 21%-25% of patients with Isaacs syndrome being diagnosed with cancer. Screening for malignancy should be performed carefully [2]. Anti-CASPR2 antibodies are associated with thymoma in 20% of cases [10]; in this case, repeated CT scans did not reveal any tumor in the course of the disease (including thymoma).

In this case, carbamazepine had a limited effect on the treatment of this patient. As for steroids, a high dose of methylprednisolone (1 g/day for three days) caused a mild improvement in his symptoms; however, when he was switched to oral prednisone (60 mg/day), his symptoms soon worsened. Although the patient, in this case, was antibody-negative, anti-VGKC complex antibodies were mainly IgG4. Plasma exchange is recommended and was effective in this case as well [8]. The first plasma exchange was effective in relieving symptoms for approximately six months; however, the duration of the effect gradually reduced such that after the third plasma exchange, side effects, such as thrombophlebitis and right internal jugular vein obstruction due to catheter placement, forced the discontinuation of the procedure.

Although IVIg was initially effective, the duration of the benefit gradually reduced and the condition worsened within less than a month. It has also been suggested that IVIg promotes Ca2+ release from the sarcoplasmic reticulum, thereby exacerbating muscle stiffness [11].

Rituximab is a widely used B-cell-eliminating monoclonal antibody. It has shown efficacy as a fast-acting targeted therapy that is proving to be effective and well-tolerated in several neuroinflammatory diseases [12]. B cells play a vital role in several autoimmune diseases, including immune-mediated neurological disorders such as neuromyelitis optica [13], multiple sclerosis [14], and myasthenia gravis [15]. Targeting B cells is considered effective for ameliorating both central and peripheral autoimmune diseases [12]. In a previous report, it was shown that rituximab was well-tolerated in the treatment of immunologic neurologic diseases, with no severe infusion reactions. Rituximab was also found to be beneficial in the treatment of immunologic neurologic disease for seven years [16].

We treated the patient with rituximab to suppress the production of unknown antibodies from B lymphocytes over a long period. In this patient, rituximab resulted in prolonged symptomatic relief without side effects. Rituximab treatment with strong and prolonged suppression of antibody production from B lymphocytes was safer and more effective than repeated plasma exchange and IVIg in suppressing symptoms for a longer period.

The limitation of this case report is that the etiology of this patient's Isaacs syndrome remained unclear because the patient was negative for CASPR2 and LGI1 antibodies. Therefore, although there are reports of treated cases, such as the CASPR2 antibody-positive case [9], the treatment of the antibody-negative patient with Isaacs syndrome is not detailed although there are reports of effective immunotherapy [2].

Rituximab should be considered early in the treatment of patients with antibody-negative Isaacs syndrome who are responsive to immunotherapy and whose long-term symptoms are difficult to control with corticosteroids, IVIg, plasma exchange, or other immunosuppressive agents.

## Conclusions

Herein, we report the case of a 23-year-old man who showed neuromyotonia in his left thigh muscle with severe pain due to PNH. Our suspected diagnosis, which we confirmed after treatment, was Isaac’s syndrome localized to the left leg. In this case, the patient tested negative for the CASPR2 and LGI1 antibodies. The treatment of this patient's symptoms of Isaacs syndrome with carbamazepine, IVIg, plasma exchange, steroids, and cyclosporine was partially effective but could hardly control symptoms. In the treatment of Isaacs syndrome, rituximab should be considered when symptoms frequently flare despite the patient’s response to steroids, IVIg, plasma exchange, and other immunosuppressive agents.
